# Dietary Factors and Type 2 Diabetes in the Middle East: What Is the Evidence for an Association?––A Systematic Review

**DOI:** 10.3390/nu5103871

**Published:** 2013-09-26

**Authors:** Lena Al-Khudairy, Saverio Stranges, Sudhesh Kumar, Nasser Al-Daghri, Karen Rees

**Affiliations:** 1 Division of Health Sciences, Warwick Medical School, University of Warwick, Coventry CV4 7AL, UK; E-Mails: s.stranges@warwick.ac.uk (S.S.); karen.rees@warwick.ac.uk (K.R.); 2 WISDEM Centre, University Hospitals of Coventry and Warwickshire, NHS Trust, Clifford, Bridge Road, Coventry, CV2 2DX, UK; E-Mail:sudhesh.kumar@warwick.ac.uk; 3 Prince Mutaib Chair for Osteoporosis, Biochemistry Department, College of Science, King Saud University, PO Box, 2455, Riyadh, 11451, Kingdom of Saudi Arabia; E-Mail: ndaghri@ksu.edu.sa

**Keywords:** diet, type 2 diabetes, Middle East

## Abstract

This review aims to search and summarise the available evidence on the association between dietary factors and type 2 diabetes mellitus (T2DM) in Middle Eastern populations, where diabetes prevalence is among the highest in the world. Electronic databases were searched; authors, libraries, and research centres in the Middle East were contacted for further studies and unpublished literature. Included studies assessed potential dietary factors for T2DM in Middle Eastern adults. Two reviewers assessed studies independently. Extensive searching yielded 17 studies which met the inclusion criteria for this review. The findings showed that whole-grain intake reduces the risk of T2DM, and potato consumption was positively correlated with T2DM. Vegetables and vegetable oil may play a protective role against T2DM. Dietary patterns that are associated with diabetes were identified, such as Fast Food and Refined Grains patterns. Two studies demonstrated that lifestyle interventions decreased the risk of T2DM. In summary, the identified studies support an association between some dietary factors and T2DM; however, many of the included studies were of poor methodological quality so the findings should be interpreted with caution. The review draws attention to major gaps in current evidence and the need for well-designed studies in this area.

## 1. Introduction

Diabetes mellitus is a global health burden affecting 285 million adults worldwide (6.4%) and costing the international health care system USD 367 billion [1]. It is also considered to be one of the most significant emerging public health problems in Middle Eastern countries. Global estimates have shown that the Middle East, as a whole, is ranked second in the world, among WHO regions, for the prevalence of diabetes, with an average prevalence of 9.3% [2]. Diabetes prevalence is projected to double over the next two decades in Middle Eastern countries [2,3].

The diet-diabetes relationship has received a great deal of scientific attention over the past decades, accompanied by methodological efforts to assess dietary intake accurately [4]. High caloric intake increases the risk of type 2 diabetes mellitus (T2DM) by increasing body weight, thus decreasing insulin sensitivity [5]. Refined carbohydrates, which are high in fructose, may increase the risk of T2DM by increasing insulin resistance [6]. International evidence has identified some dietary items, such as whole-grain rich foods, cereal fibre, legumes, and green leafy vegetables that play a protective role against chronic conditions including T2DM [7,8,9]. The nutritional composition (*i.e.*, fibre, vitamins and minerals) of protective foods may decrease the risk of T2DM by reducing inflammation, improving glucose metabolism, endothelial function, and insulin sensitivity [10].

The consumption of sugar sweetened beverages showed a positive association with T2DM, this association is mediated by increased body weight which disrupts glucose metabolism and insulin sensitivity [11,12]. Dietary energy density (DED) is correlated with T2DM by increasing body weight, and energy dense foods seem to increase glycaemic load and insulin resistance [13]. Examining the diet as a whole in relation to health outcomes has complemented the traditional single nutrient assessment [14]. Studies have identified some protective dietary patterns against T2DM, such patterns are characterized by high intakes of vegetables, fruits, whole-grains and legumes [15,16]. Dietary patterns with high consumptions of processed meats, refined grains, sugar-sweetened beverages and fatty foods seem to increase the risk of diabetes [17,18].

It is well-established that lifestyle and dietary interventions play a major role in the prevention of T2DM both in the general population and high-risk individuals, but this evidence comes mostly from Western populations [19,20,21,22,23]. Diet-related chronic conditions represent a major public health concern in the Middle East [24,25,26]. The rapid urbanization and the fast economic boost imported the “Western diet” into the Middle East. The nutritional transition in the Middle East introduced energy-dense, refined carbohydrates and fat-saturated cuisine [27]. This transition has paralleled the increase in lifestyle-related chronic conditions, such as diabetes [28,29]. However, evidence linking these dietary habits to the emerging diabetes epidemic is not clearly defined in these settings [30,31,32,33]. Therefore, we aimed to perform a systematic review of the literature to summarise the available evidence on the association between diet and the risk of T2DM in Middle Eastern countries, and to identify gaps for further research.

## 2. Materials and Methods

MEDLINE, CINAHL, Web of Science, and the Cochrane Library were searched from inception until May 2013. Reference lists of retrieved articles were scanned for further studies, and a registration of electronic email updates for relevant new published articles was performed. Grey literature was scrutinised (Saudi Bureau Library-UK) using electronic databases and hand searching of relevant theses. Authors, libraries (The British Library-UK, King Abdul-Aziz City of Science and Technology-KSA), and research centres (King Faisal Specialist Hospital & Research Centre in Jeddah and Riyadh-KSA, Bahrain Centre for Studies and Research) were contacted for further studies and unpublished literature. No restrictions were made by language or year of publication. The search terms used included medical subject headings (MeSH) or the equivalent, and text word terms (*i.e.*, diet, diet records, nutrition/diet surveys, nutrition assessment, eating, food habits, AND Middle East AND type 2 diabetes mellitus). Middle Eastern countries were based on MEDLINE list of countries. A specialist librarian was consulted for further search terms. Searches were tailored to individual databases.

Studies met the inclusion criteria if they fulfilled all of the following criteria:

Study design—randomised controlled trials, non-randomised intervention studies, cohort studies, case-control studies or cross-sectional studies;

Participants—all Middle Eastern adults (≥18 years of age) with the exception of those with type 1 diabetes;

Exposure/intervention—nutrition or dietary variables, including the quality/quantity of food intake, interventions aimed at dietary changes, food habits or dietary patterns measured via nutritional tools, or nutritional biomarkers; and

Outcome—incidence or prevalence of T2DM.

Following the database searching, titles and abstracts of articles were screened for potential relevance by one reviewer (LA). Studies not carried out in the Middle East, studies of children, and studies not measuring diabetes were excluded at this stage. Following this preliminary screening, full reports of potentially relevant studies were obtained, and two reviewers (LA, KR) independently assessed studies for inclusion/exclusion using a checklist form based on the four inclusion criteria above. Where there was disagreement about the inclusion of a study, a third reviewer was consulted (SS).

Data were extracted from the included studies by two reviewers (LA, KR) independently using a predefined data abstraction form. Key data including details of the study design, participant characteristics, study setting, intervention/exposure (including assessment/validation, potential confounders), risk of bias (selection of participants, losses to follow up), and outcome assessment/method of diagnosis were extracted from each of the studies that met the inclusion criteria.

## 3. Results

**Figure 1:**
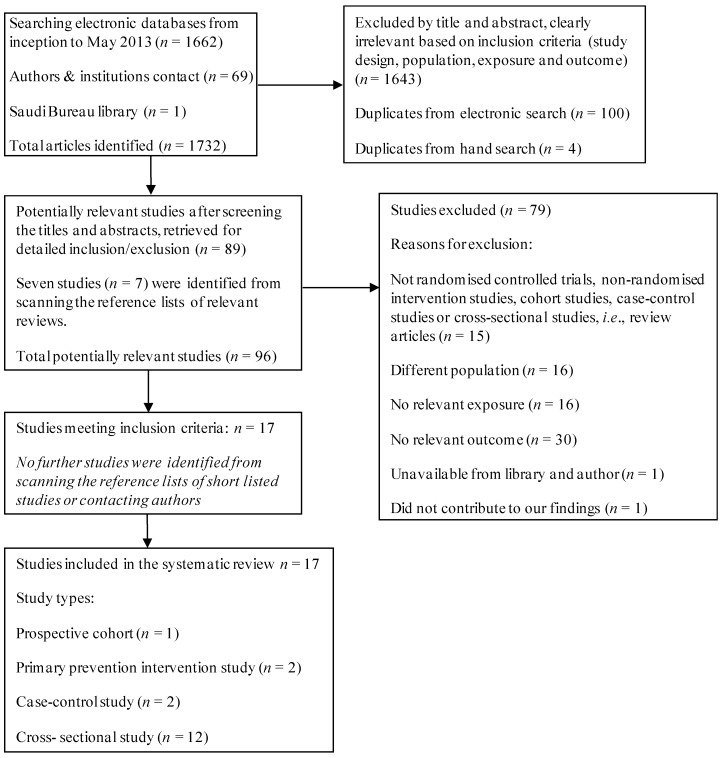
Flow diagram for the selection of studies.

Searching the electronic databases yielded 1662 references. Contacting authors, research centres, and searching the grey literature yielded an additional 69 references. Reading the titles and abstracts for potential relevance excluded 1643 articles, as they did not meet the above mentioned inclusion criteria of this review (see Figure 1), leaving 89 potentially relevant articles. Seven further studies were identified from scanning the reference lists of the 89 short-listed studies. In total, 96 studies went forward for formal inclusion/exclusion. Seventeen studies met the inclusion criteria, while the remaining 79 studies were excluded due to irrelevant study design, participants, exposure and outcome (see Figure 1).

A narrative analysis was chosen as the most appropriate method to analyse the data, as the included studies were heterogeneous. The included studies used different study designs and measured different exposures/interventions. Details of the included studies are shown in Table 1, Table 2 and Table 3. A total of 17 studies met the inclusion criteria for this systematic review.

**Table 1:** Association of energy, nutrients, foods, and beverages with T2DM.

Author	Study design	Country/study population	Sample size	Sex (%)	Age (years)	DietaryAssessment method	Dietary factor	Results
**Energy and nutrients**								
Kahn *et al*., 1971 [34]	Prospective cohort. Follow-up: 2 years	Israeli civil-services employees	8369	M: 100	>40	Short dietary questionnaire	Total calories (kcal/day), total carbohydrate (g/day), animal protein (g/day), saturated fatty acid (g/day), and sugar calories (kcal/day).	There was no association between dietary variables assessed and T2DM incidence.
**Foods**								
Midhet *et al.*, 2010 [35]	Case-control	Saudi Arabian PHCC’s attendees	498	M: 48.6 F: 51.4	30–70	Food preference questions and 24-h DR	Food items consumed regularly, Kabsa (rice/chicken with rice), dates, fish, vegetables, bakery items, potato chips and/or French fries, snacks and hummus, full fat dairy products, coffee and/or tea with sugar, juices and soft drinks.	Routine consumption of Kabsa (OR 5.5, CI: 2.3–13.5), bakery items (OR 2.4, CI: 1.3–4.6), French fries (OR 2.2, CI: 1.2–3.9) and fish (OR 2.5, CI: 1.3–4.7) were associated with an increased risk of T2DM. Vegetables showed a protective effect (OR 0.4, CI: 0.2–0.7).
Ezmaillzadeh *et al.*, 2005 [36]	Cross-sectional	Iranian residents	827	M: 43.2 F: 56.8	18–74	Validated 168-items FFQ (Willet format)	Whole-grain foods (e.g., dark breads, barley bread, popcorn, whole-grain breakfast cereal, wheat germ and bulgur). Refined grain foods (e.g., white breads, iceberg bread, noodle, pasta, rice, toasted bread, milled barley, sweet bread, white flour, starch and biscuits).	The highest quartile of whole-grain consumption was associated with a reduced risk of T2DM (OR 0.88, CI: 0.8, 0.94) as compared to the reference category (*p* < 0.05). There was no significant increase in the risk of diabetes with refined-grain consumption (highest category OR 1.14, CI: 0.87–2.52)
Esmillzadeh *et al*., 2011 [37]	Cross-sectional	Iranian teachers	486	F: 100	≥40	Validated 168-items FFQ (Willet format)	Vegetable oil which included partially hydrogenated vegetable oil (PHVO) and non-hydrogenated vegetable oil (NHVO) (e.g., soyabean oil, olive oil, sunflower oil, maize oil, rapeseed oil).	No significant association was found between PHVO (*p* = 0.31) or NHVO (*p* = 0.19) and diabetes. However, diabetes prevalence increased across PHVO quintiles, and decreased across NHVO quintiles.
Khosravi-Boroujeni *et al*., 2012 [38]	Cross-sectional	Iranian residents	4774	M: 76 F: 24	>19	Validated 49-items FFQ	Potato consumption.	There was a positive association (*p* < 0.001) between potato intake and risk of diabetes (OR 1.38, CI: 1.41–1.67).
**Beverages**								
Golozar *et al*., 2011 [39]	Cross-sectional	Iranian residents	50,039	M: 42.4 F: 57.6	≥30	Validated 158-items FFQ	Green and black tea consumption (mL/day).	Heavy green tea consumption (≥600 mL/day) was positively associated with T2DM (prevalence ratio (PR) 1.24, CI: 1.05–1.47)
**Energy density**								
Esmillzadeh *et al*., 2012 [40]	Cross-sectional	Iranian teachers	486	F: 100	≥40	Validated 168-items FFQ (Willet format)	Dietary energy density (DED) from food (kcal/g) ^1^.	No significant association between the highest quartile of DED_Food_ (prevalence ration (PR): 1.06, CI: 0.42–2.73) and diabetes.
Kalter-Leibovici *et al*., 2012 [41]	Cross-sectional	Israel (Jewish and Arab residents)	1092	M: 49.6 F: 50.4	≥25	240-items FFQ	DED_Food + Beverages _(kcal/g) ^2^	Arabs with diabetes were more likely to be in the highest quartiles of DED (29.5% *vs.* 35.4%). The risk of diabetes was significantly higher in highest quartiles of DED (adjusted hazards ratio: 1.67, CI: 1.08–2.61) in comparison to lower quartiles (adjusted hazards ratio: 1.53, CI: 0.98–2.39).

FFQ: Food frequency questionnaire. 24-h DR: Twenty-four hour dietary recall. PHCC’s: Primary health care centres. T2DM: Type 2 diabetes mellitus. PHVO: Partially hydrogenated vegetable oil. NHVO: Non-hydrogenated vegetable oil. ^1^ DED was calculated by: energy intakes from foods (kcal/day)/total weight of foods consumed (g/day). ^2^ DED was calculated by: total energy intake (kcal/day)/total weight of food and drinks consumed (g/day).

**Table 2:** Association between dietary patterns and T2DM.

Author	Study design	Country/study population	Sample size	Sex (%)	Age (years)	Dietary	Dietary factor	Results
						Assessment method		
***A priori* Dietary Patterns**								
Bilenko *et al*., 2005 [42]	Cross-sectional	Israeli residents	1159	M: 44.9 F: 55.1	≥35	24-h DR	Mediterranean dietary score ^1^.	No significant difference was observed across Mediterranean diet score categories (low or high) and the prevalence of diabetes in both males and females.
Azadbakht *et al*., 2006 [43]	Cross-sectional	Iranian residents	581	M: 51 F: 49	≥18	Validated 168-items FFQ (Willet format)	Dietary diversity score (DDS) ^2^, which was from the five main food groups of the Food Guide Pyramid (bread/grains, fruits, vegetables, dairy, meat and meat substitutes). The five groups were divided into 23 (e.g., vegetables: vegetables, potatoes, tomatoes, starchy vegetables, legumes, yellow vegetables, green vegetables).	Although there was no protective effect of healthier diet score against diabetes, the risk of diabetes decreased significantly across quartiles of DDS (*p* = 0.03). Quartiles of DDS for whole-grains (OR-Q_1_ 1.45, CI: 1.09-1.88 *vs.* OR-Q_3_ 1.11, CI: 0.89–1.44), and vegetables (OR-Q_1_ 1.12, CI: 0.54–1.88 *vs.* OR-Q_3_ 1.05, CI: 0.89–1.34) did not have an inverse association with diabetes.
Naja *et al*., 2012 [44]	Case-control	Lebanon (cases: Lebanese private clinic attendees, controls: Lebanese residents)	174	M: 60.3 F: 39.7	>18	97-items FFQ	4 dietary patterns, Refined Grains and Desserts (e.g., pasta, pizza, deserts), Traditional Lebanese (e.g., whole wheat bread, olives and olive oil), Fast Food (e.g., mixed nuts, French fries, and full fat milk), and Meat and Alcohol patterns (e.g., red meat, eggs, carbonated beverages).	The Traditional Lebanese pattern showed significantly lower odds of T2DM (OR 0.46, CI: 0.22–0.97) while the Refined Grains (OR 3.85, CI: 1.31–11.23) and the Fast Food patterns (OR 2.80, CI: 1.41–5.59) significantly increased the odds of T2DM in Lebanese adults.
Esmillzadeh *et al*., 2008 [45]	Cross-sectional	Iranian teachers	486	F: 100	≥40	Validated 168-items FFQ (Willet format)	3 dietary patterns, Healthy (e.g., fruits, vegetables, legumes), Western (e.g., red meat, butter, pizza), and Iranian patterns (e.g., refined grains, potato, broth).	The prevalence of diabetes decreased significantly among quintiles of Healthy pattern (*p* < 0.05) and increased among quintiles of Western (*p* < 0.05) and Iranian patterns (*p* = 0.24). The Healthy pattern had a protective effect against diabetes (OR 0.29, CI: 0.11–1.07, *p* = 0.07).
Abu-Saad *et al*., 2012 [46]	Cross-sectional	Israel (Jewish and Arab residents)	1104	M: 50 F: 50	≥25	240-items FFQ	4 dietary patterns, Ethnic (e.g., pita bread, olive oil and Arabic mixed meat), Healthy (e.g., fruits, low fat dairy products and whole grains), Fish and Meat Dishes (fish, meat and frying oil), Middle Eastern snacks and Fast Food patterns (e.g., savoury cheese, nuts, and fast food).	Scores for the Healthy and Ethnic pattern clearly differed by ethnicity. Hence, the two patterns were used for further analysis. The prevalence of diabetes was higher in increased tertiles of Ethnic pattern (T_3_ 20% *vs.* T_1–2_ 13%, *p* = 0.001), and participants with prevalent diabetes were more likely to be in the highest tertiles of Healthy pattern (T_3_ 25% *vs.* T_1–2_ 10%, *p* < 0.001). Arabs with prevalent diabetes were more likely to be in the highest tertiles of the healthy pattern (OR 5.00, CI: 2.92–8.55) in comparison to Jews with diabetes (OR 2.00, CI: 1.01–3.95).
**Other Dietary Patterns**								
Al Ali *et al*., 2011 [47]	Cross-sectional	Syrian residents	1168	M: 47.7 F: 52.3	≥25	Frequency questionnaire	Healthy and unhealthy diets ^3^.	Frequent fruit and vegetable consumption was associated with a reduced risk of T2DM (OR 0.70, CI: 0.48–1.03), but this did not reach statistical significance.
Alrabadi *et al*., 2013 [48]	Cross-sectional	Jordanian residents	286	M: 49 F: 51	>40	Questionnaire	Vegetarianism ^4^.	The prevalence of diabetes was significantly lower among vegetarians (38%) in comparison to non-vegetarians (44%).

24-h DR: Twenty-four hour dietary recall. FFQ: Food frequency questionnaire. ^1^ MD scores: Reported foods (*n* = 2200) were categorized according to their dietary components (e.g., legumes, meat, vegetables and fruits) and points were given to the consumption of each group following Trichopouplou *et al*. methods [49]. The lower the score (≤4) the lower the consumption of the Mediterranean diet. ^2^ DDS scores were based on the following: (servings/subgroups) × 2. Scores were divided into quartiles and the higher the score the healthier the diet. ^3^ Diets were based on the frequency (days/week) of fruits and vegetables intake (<3 or 3–6 or 7 days/week), lower frequencies (<3) scored less (1 point), and higher frequencies (3–6, 7 days/week) scored more (2 and 3 points respectively). Participants with an unhealthy diet had lower tertiles for total scores. ^4^ Vegetarianism: A vegetarian diet was defined as meat and poultry intake <1 time/month, while a non-vegetarian diet was defined as red meat or poultry intake ≥1 time/month.

**Table 3:** Association between lifestyle factors and T2DM in intervention studies.

Author	Study design	Country/study population	Sample size	Sex (%)	Age (years)	Follow-up (Years)	Intervention	Results
Harati *et al.*, 2010 [50]	Primary prevention intervention study	Iranian residence	8212	M: 41 F: 59	>20	3.6	Intervention: At baseline: intensive education to increase physical activity, reduce cigarette smoking, and face-to-face educational interviews to improve nutritional habits. The lifestyle modification intervention was based on guidelines recommendations by The American Heart Association and modified to suit the Iranian knowledge, attitude and practice that were assessed in a previous study (KAP study) [51]. Dieticians providing tailored nutrition interventions such as weight reduction diet, exchange list education, diet management, DASH diet, and ADA nutrition principles. For the next 2.6 years: nutritional classes (held for 4 days/week at clinics), group meetings, public sites, publications, public conferences, distribution of education materials and school-based programs were carried out. Control: Did not receive the intervention.	The lifestyle modification programme resulted in a statistically significant relative risk reduction of 65% in the incidence of diabetes (95% CI = 30%, 83%, *p* = 0.003). The incidence of diabetes was 8.2 per 1000 person-years in the intervention groups in comparison to 12.2 per 1000 person-years in the control group.
Sarrafzad-egan *et al*., 2013 [52]	Primary prevention intervention study	Iranian residence	12,514 baseline (2001–2002) 9570 post-intervention (2007)	M: 50 F: 50	≥19	4	Intervention: interventions began at different times throughout the study and were at a community level using different approaches (e.g., mass media, health services). Improve healthy eating, increase physical activity, reduce tobacco smoking and cope with stress. Additional secondary preventative measures were delivered to high-risk individuals (e.g., people with diabetes). Projects were tailored to meet participants needs (e.g., Healthy-lifestyles for High-risk Populations, Healthy Food for Healthy Communities, Isfahan Exercise Project, and Healthy Lifestyles for High-risk Populations). Control: Did not receive the intervention.	The prevalence of diabetes did not decrease in the intervention group in both females (2001: 6.8%, 2007: 7.1%, *p* = 0.38) and males (2001: 5.8%, 2007: 7.1%, *p* = 0.17). However, there was a borderline significant increase in males of the reference group (2001: 4.0%, 2007: 5.7%, *p* = 0.056), and a non-significant increase in females of the reference group (2001: 5.8%, 2007: 7.3%, *p* = 0.15).

KAP study: Knowledge, Attitude and Practice study. DASH: Dietary approach to Stop Hypertension. ADA: American Diabetes Association.

### 3.1. Association of Energy, Nutrients, Foods, and Beverages with T2DM

In total, eight studies examined the association between energy, nutrients, different foods, and beverages and T2DM. For full details of the studies please refer to Table 1.

#### 3.1.1. Energy and Nutrients

A prospective cohort study [34] examined the association between total energy and some nutrients and the incidence of T2DM. Trained nurses assessed the frequency, amount and portion size of some food items (e.g., meat, poultry, fish, milk, beverages). The type and amount of fat consumed was assessed with more emphasis (e.g., what type of fat or oil used in the preparation of food).The study reported no association between the reported dietary items and the incidence of T2DM.

#### 3.1.2. Foods

A case-control study conducted in Saudi Arabia [35] measured the association between specific dietary items and the risk of T2DM. In 283 cases and 215 controls, results showed that routine consumption of certain foods such as Kabsa (OR 5.5, CI: 2.3–13.5), bakery items (OR 2.4, CI: 1.3–4.6), and French fries (OR 2.2, CI: 1.2–3.9) increased the risk of T2DM. Notably, fish consumption was associated with an increased risk of diabetes (OR 2.5, CI: 1.3–4.7). Routine consumption of vegetables showed a protective effect for the risk of T2DM (OR 0.4, CI: 0.2–0.7).

The cross-sectional study [36] examined the association between whole and refined grains and T2DM. Participants reported higher intakes of refined grains rather than whole-grains. The highest quartile of whole-grain consumption was associated with a reduced risk of T2DM (OR 0.88, CI: 0.8–0.94) as compared to the reference category (*p* < 0.05), but there was no statistically significant trend over the four quartiles. There was no significant increase in the risk of diabetes with refined-grain consumption (highest category OR 1.14, CI: 0.87–2.52).

A cross-sectional study [38] examined the association between dietary potato intake (boiled form) and T2DM prevalence in Iranian adults. The results showed a positive association between the frequency of potato intake (>once/week) and T2DM (OR 1.38, CI: 1.14–1.67, *p* < 0.001).

A cross-sectional study of Iranian female teachers [37] examined the correlation between vegetable oil and T2DM prevalence. Women had a higher intake (23 ± 11 g/day) of partially hydrogenated vegetable oil (PHVO) in comparison to non-hydrogenated vegetable oil (NHVO) 22 ± 10 g/day. The prevalence of diabetes increased among quintiles of PHVO, however, no significant associations were observed between diabetes and PHVO (OR: 2.11, CI: 0.55–9.47) or NHVO (OR: 0.51, CI: 0.10–1.51).

#### 3.1.3. Beverages

A further cross-sectional study [39] assessed the association between tea consumption and the prevalence of T2DM in Iranian adults. Heavy green tea consumption (≥600 mL/day) was associated with T2DM (PR 1.24, CI: 1.05–1.47) while no significant association was observed for black tea (PR 1.02, CI: 0.94–1.12).

#### 3.1.4. Energy Density

The first study [40] observed the association between DED_Food_ and T2DM in Iranian women. Fasting plasma glucose increased among quartiles of DED_Food_, however, no significant associations were observed between higher DED_Food_ and diabetes (PR: 1.06, CI: 0.42–2.73).

The second study [41] assessed the association between DED_Food + Beverages_ and T2DM in both Arabs and Jews. There was no significant association between DED_Food + Beverages_ and diabetes (*p* = 0.08). However, participants with higher DED_Food + Beverages_ (≥0.886) had a higher risk of diabetes (Adjusted hazard ratio: 1.67, CI: 1.08–2.61).

### 3.2. Association between Dietary Patterns and T2DM

In total, seven studies assessed different dietary patterns in relation to T2DM. For full details of the studies please refer to Table 2.

#### 3.2.1. *A Priori* Dietary Patterns

The first study [43] assessed the association between dietary diversity score (DDS) and T2DM in Iranian adults. Trained dieticians assessed dietary intake. The results showed that the probability of having diabetes decreased among increasing quartiles of DDS (*p* = 0.03). A decrease of diabetes probability was observed among quartiles of whole grains DDS (OR-Q_1_ 1.45, CI: 1.09–1.88 *vs.* OR-Q_3_ 1.11, CI: 0.89–1.44). However, there was no observed protective effect of DDS of vegetables against diabetes (OR_vegetables_ 1.05, CI: 0.89–1.34).

The second study [42] examined the association between the Mediterranean diet score (MD) and the prevalence of T2DM. Reported food groups were given a score (≤4 = low MD or ≥5 = high MD) to categorise the intake of the Mediterranean diet. The results showed no statistical differences for the prevalence of diabetes across high or low consumers of the MD. The prevalence of diabetes in males with low consumption of the MD was 12.7% while high consumers had a prevalence of 11.0%. A similar trend was found in females (13.6% *vs.* 9.3% respectively).

#### 3.2.2. *A Posteriori* Dietary Patterns

A case-control study conducted in Lebanon [44] measured the association between diet and T2DM. Dietary intake was assessed in one to one interviews using a FFQ that represented the Lebanese diet. Principal component factor analysis (PCA) identified four dietary patterns, Refined Grains and Dessert, Traditional Lebanese, Fast Food, and Meat and Alcohol patterns. In 58 newly diagnosed cases and 116 controls, results showed that Refined Grains and Desserts (OR 3.85, CI: 1.31–11.23), and Fast Food (OR 2.80, CI: 1.41–5.59) patterns significantly increased the risk of T2DM. Conversely, the Traditional Lebanese pattern had a protective effect (OR 0.46, CI: 0.22–0.97). No association was observed between the Meat and Alcohol pattern and T2DM (OR 1.43, CI: 0.83–2.46).

A cross-sectional study [45] which included female teachers from Tehran, Iran, examined dietary patterns in relation to T2DM prevalence. PCA identified three dietary patterns, Western, Healthy and Iranian. The prevalence of diabetes decreased among quintiles of Healthy pattern (*p* < 0.05) and increased among quintiles of the Western pattern (*p* < 0.05). There were no significant associations between the prevalence of T2DM and quintiles of the Iranian pattern (*p* = 0.24). The Healthy pattern had a borderline significant protective effect against diabetes (OR 0.29, CI: 0.11–1.07, *p* = 0.07), while no significant associations were observed for the Western and Iranian dietary patterns (OR 3.42, CI: 0.88–13.31, OR 2.11, CI: 0.66–7.12 respectively).

A further study [46] assessed the association between dietary patterns and T2DM. PCA identified four dietary patterns, Ethnic, Healthy, Fish and Meat, Middle Eastern snacks and fast food patterns. Participants with diabetes were more likely to be in higher tertiles of the Healthy pattern (T_1–2_ 10% *vs.* T_3_ 25%, *p* < 0.001)_._ Arabs with diabetes were more likely to be in higher tertiles of the Healthy pattern in comparison to Jews (OR: 5.00, CI: 2.92–8.55 and OR: 2.00, CI: 1.01–3.95 respectively).

#### 3.2.3. Other Patterns

A cross-sectional study [47] examined the association between unhealthy diet scores and the prevalence of T2DM in Syrian adults. The questionnaire assessed the frequency of fruit and vegetable consumption. Results showed that having an unhealthy diet (lower frequencies of fruits and vegetables consumption) did not show a significant protective effect against T2DM (OR 0.70, CI: 0.48–1.03).

A cross-sectional study [48] assessed the association between a vegetarianism and the prevalence of diabetes in Jordanian adults. Participants were required to complete a questionnaire to report whether they were vegetarians or non-vegetarians. Diabetes prevalence was significantly higher (*p* < 0.05) among non-vegetarians (44%) in comparison to vegetarians (38% reported diabetes).

### 3.3. Association between Lifestyle Factors and T2DM

In total, two non-randomised primary prevention studies examined the effect of lifestyle interventions on T2DM in Iranian adults. For full details of the studies please refer to Table 3.

The first study [50] assessed the effectiveness of a multi-factorial intervention on the incidence of T2DM in the community. One cluster (from one health centre, *n* = 3098) received the intervention, whilst two clusters (from two health centres, *n* = 5114) acted as the control group. The incidence of diabetes was 12.2 per 1000 person-years in the control group, and 8.2 per 1000 person-years following the intervention, with a relative risk reduction of 65% (95% CI = 30%–83%, *p* = 0.003). The attributable risk reduction (ARR) was 0.39% and the number needed to treat (NNT) was 256 to prevent one case of diabetes in the whole population.

The other intervention study [52] examined the effect of comprehensive lifestyle modification in Iranian adults for nearly four years. The baseline sample (2000–2001) included 6175 participants in the intervention group and 6339 participants in the control group. Post lifestyle modification (2007) included 4179 participants in the intervention group while the control group included 4853 participants. The baseline prevalence of diabetes in the intervention area was 6.8% in females and 5.8% in males. The comprehensive lifestyle program did not reduce the prevalence of diabetes in the intervention area (7.1% in females and 7.1% in males). However, the prevalence of diabetes in the reference area increased by 25.9% in females and 42.5% in males.

## 4. Methodological Quality of Included Studies

The assessment of the methodological quality of the included studies (all non-randomised) was informed by guidelines from the Cochrane Handbook [53]. The methodological quality of the prospective cohort study [34] and the case-control studies [35,44] were assessed using the Newcastle-Ottawa Quality Assessment Scale [54]. Results are presented in Table 4.

**Table 4:** Methodological quality of the cohort study and case-control studies.

Cohort Study	Selection	Comparability	Outcome
Kahn *et al.* [34]	**	*	*
**Case-control Study**	Selection	Comparability	Exposure
Midhet *et al.* [35]	****	**	*
Naja *et al.* [44]	****	**	**

The Newcastle-Ottawa Assessment Scale [54] for the cohort studies—a study can be awarded a maximum of one star for each category of selection (representative of the exposed cohort, selection of the non-exposed cohort, ascertainment of exposure), and outcome categories (assessment of outcome, sufficient follow-up for outcome to occur, adequacy of cohorts follow up). A maximum of 2 stars can be awarded for comparability (controls for important factors, controls for additional factors). Case-control studies—a study can be awarded a maximum of 4 stars for selection (case definition, representativeness of the cases, control selection and definition of the controls), a maximum of 2 stars for comparability (cases and controls must be matched for in the design of analysis) and a maximum of 4 stars for exposure (three questions assessing ascertainment of the exposure, the same method of ascertainment of exposure in cases and controls, and the non-response rate).

The prospective cohort study [34] included male-civil service employees working in three different cities in Israel so they were a selected group and not truly representative of the Israeli population. Trained nurses administered the dietary questionnaire and models of foods were used to estimate portion size. More emphasis was given to the type and amount of fat consumed, which may have introduced some bias. Participants with diabetes or abnormal glucose levels were excluded from the recruitment. The association between dietary variables and diabetes were not clearly presented in this study, however, analysis was stratified by age groups and place of birth so this study scored one of a possible two stars for comparability. The assessment of diabetes was unclear and losses to follow up were not described, but the follow up period of two years was sufficient as 131 incident cases of T2DM were identified, hence one star was awarded for the outcome.

In the case-control study by Midhet and colleagues [35], cases were known to the primary health care centres (PHCC’s), and controls were selected on the basis of an absence of diabetes (blood glucose level of ≤180 mg/100 mL) from the same population. Every fifth patient visiting the PHCC’s was asked to participate in the study. The authors state that the cases and controls were not matched, but potential confounders (including age, sex, and family history of diabetes) were controlled for in the analysis. Interviewers were not blinded to cases or controls, and cases were asked more questions on their dietary habits prior to the diagnosis of T2DM, which may increase the likelihood of assessor’s bias and recall bias. The exposure was specific food items, with no justification by the authors for the chosen foods, and the nutritional composition of foods was not examined in this study. The authors state that some participants with incomplete data were excluded from the analysis, but the numbers were not provided separately for the cases and controls.

The case-control study by Naja and colleagues [44] included cases that were newly diagnosed with T2DM (within six months) attending private Clinics or the Dietary Department at the American University of Beirut Medical Centre. Healthy controls (free of self-reported diabetes) were from the general population living in the same residential area as the cases. Two stars were awarded for comparability; two controls were selected to match each case, by age, gender and residential area. The FFQ was not validated, and the interviewers were not blinded to cases or controls. Cases were asked to report dietary intake one year before diabetes diagnosis while controls were asked to report for the previous year. The foods assessed by the questionnaire were clearly mentioned and nutritional analysis was clearly described. The response rate of cases was 89% and 82% for controls.

The methodological quality of the primary prevention intervention studies [50,52] was assessed using the Cochrane risk of bias tool [53]. This tool examines six domains, namely: sequence generation, allocation concealment, blinding of participants, personnel and outcome assessors, incomplete outcome data, selective outcome reporting and other sources of bias. Neither of the two intervention studies was randomised so the first two domains of the risk of bias tool are not relevant here. The study by Harati and colleagues [50] had a clustered design whereby one medical centre (of a total of 20 enrolled in a large prospective study) received the intervention, and two acted as the comparison group. Blinding of lifestyle interventions is problematic, but this was addressed to some degree in the cluster design. The primary outcome was incidence of T2DM, which was clearly defined. Possible confounding variables were adjusted for in the analysis, and clustering was taken into consideration by the use of a random effects model. The loss to follow-up was high at 40%, but this was comparable between intervention and comparison groups.

The Sarrafzadegon *et al.* study [52] had a clustered design where two districts served as the intervention group, and one district served as the control. Diabetes was one of the cardiometabolic risk factors assessed in the study, and a diagnosis of diabetes was confirmed if FPG ≥ 126 mg/dL or the intake of diabetes medications. The outcomes of the study were examined in two cross-sectional surveys, one in 2000–2001 (baseline) and one in 2007 (post intervention) and participants were selected using multistage random sampling.

The methodological quality of the cross-sectional studies [36,37,38,40,43,45,47,48] was assessed by means of choice of population, sampling method, exposure, and outcome measurement. The population of two studies [36,43] were a subsample of a larger prospective study (TLGS) which included 15,005 participants from different age groups and genders living in Tehran. The authors state that the participants were randomly selected. Dietary intake was assessed using a validated FFQ (against twelve 24-h dietary recalls and biomarkers) to measure participant’s intake of whole-grain and refined grain, and to assess the dietary diversity score (DDS). Dieticians completed the FFQ. The foods assessed were clearly defined, as were the methods of DDS calculation. Outcome measures were robust and clearly defined (FPG ≥ 126 mg/dL or 2 OGTT ≥ 200 mg/dL).

Three cross-sectional studies by Esmaillzadeh *et al.* [37,40,45] were conducted in the same population but reported different dietary exposures. The population studied was limited to female teachers working in Tehran, Iran. The authors used multistage cluster random sampling method, where they divided the 20 districts of Tehran Educational Offices into four areas (northern, southern, western, and eastern). One district from each area was randomly selected, followed by a random selection of schools (private and public) and number of teachers for each district. The dietary assessment tool (FFQ) was validated against twelve 24-h dietary recalls and recovery biomarkers. The FFQ was used to look at different dietary factors, vegetable oils, DED_Food_, and dietary patterns in relation to diabetes. The two vegetable oils assessed in the study (PHVO and NHVO) were clearly defined. The methods of DED_Food_ calculations were clearly defined by the authors. Although the authors calculated DED from food only, they did not adjust for energy intake from beverages. The authors clearly stated the food grouping method used for dietary patterns that was based on nutrients similarity. The labelling of each identified pattern and the foods included were clearly mentioned in the study. Diabetes diagnosis was well defined (FPG ≥ 6.93 mmol/L).

The study by Khosravi-Boroujeni and colleagues [38] included a sub-sample Isfahan Healthy Heart Programme-2003 (*n* = 12,514). Multistage random-cluster sampling method was used. Trained technicians completed a FFQ to assess potato intake. The authors state that the FFQ was validated, however, methods of validation were not clearly stated. The outcome was clearly defined (FPG > 126 mg/dL or 2-h postprandial > 200 mg/dL or use of diabetes drugs).

The population of the Golozar *et al.* study [39] is part of the Golestan Cohort Study (*n* = 50,044) which included both genders from different age groups. Systematic clustering based on household numbers was used to randomly recruit 39,399 residents in the Golsestan Province. The remaining participants, in rural areas, were contacted through the primary health care centres in villages. Trained dieticians administered a FFQ to assess tea intake. The questionnaire was validated against twelve 24-h recalls in 131 individuals. The authors did not justify the choice of dietary variables. Diabetes was self-reported.

Two cross-sectional studies from Israel [41,46] included a random population-based sample from Hedra district. A random sample was selected from the population registry and was equally stratified by gender and ethnicity. The sample had similar socio-demographic characteristics. Personal interviews were carried out using a 2-step quantified FFQ. The FFQ was developed for Jewish people and modified for Arabs. However, the questionnaire was not validated. DED_Food + Beverage_ and dietary patterns were assessed. DED calculation was clearly defined, however, caloric consumption reports of 6000 kcal/day were included in the analysis, and it is possible that outliers and over-reporters increased the distribution of the data and affected the overall dietary results. The labelling of each dietary pattern and the foods included were clearly mentioned. The food items were grouped based on similarity of common usage, ethnic origin and nutrient composition. Potential confounders were clearly adjusted for in the analysis. Diabetes was self-reported.

The cross-sectional study by Bilenko *et al.* was part of the Negev Nutrition Study in Israel [42]. A random proportional geographic cluster sample of the Negev residence was selected, and a random adult of each household was chosen to participate in the study. A single modified multi-pass 24-h questionnaire, that was adapted from the United States Department of Agriculture, was used to collect dietary information. Although a single recall cannot represent usual intake, the multi-pass method provides a structured and staged interview that allows the participant to recall dietary information. The Mediterranean scoring methods were clearly defined. Diabetes was not the main outcome in the study and possible confounders were not adjusted for in the analysis. Outcome measures were clear in the study.

The household survey by Al Ali *et al.* [47] was part of the 2nd Aleppo Household Survey conducted in 2006. Two-stage cluster sampling method was used, 27 neighbourhoods were randomly selected, followed by a random selection of 1268 households, and a random adult (≥25 years) from each household was invited to participate. Interviewers administered a questionnaire that assessed the frequency of fruit and vegetable intake. Dietary methods indicate that the frequency categories of fruits and vegetables were based on arbitrary decisions. Diabetes diagnoses was based on self-reports and FPG ≥ 126 mg/dL.

The cross-sectional study [48] conducted in Ajloun, Jordan did not report the questionnaire distribution, population choice, and sampling methods. The authors did not justify the categorization of vegetarians (*i.e.*, what guidelines they followed, as some participants labelled as vegetarians ate meat but infrequently). Participants completed the questionnaires, and diabetes was self-reported.

## 5. Discussion

Extensive searching including electronic databases, hand searching of relevant theses, libraries, research centres, and contacting authors in the field, identified relatively few studies reporting an association between dietary factors and the risk of T2DM in Middle Eastern adults. Seventeen studies met the inclusion criteria, one prospective cohort [34], two primary prevention intervention studies [50,52], two case-control [35,44], and 12 cross-sectional studies [36,37,38,39,40,41,42,43,45,46,47,48]. Although some of the included studies assessed similar nutritional exposures, they were of different methodology and study design.

One of the very few cohort studies in the Middle Eastern area found no association between total energy and nutrient intake and the incidence of T2DM in men [34]. Nutritional evidence provides controversial findings on the associations between dietary macronutrients and diabetes [55]. However, large cohort studies have highlighted the effect of types of dietary fat [56], protein [7], and carbohydrates [57] on the risk of T2DM. Ezmaillzadeh and colleagues [36] reported an inverse association between whole-grain consumption and the risk of diabetes. These findings do parallel results of observational studies from US populations [58,59]. For example, the Framingham Offspring Study found that whole-grain consumption improved metabolic markers in 3481 participants, thus reducing the risk of T2DM [58]. Similarly, the Nurses’ Health Studies (NHSs) I and II found that whole-grain intake had a protective role against T2DM [59].

In this review, two studies reported an association between increased potato consumption and risk of T2DM [35,38]. The effect of residual confounding is possible when examining single foods, and adjustment for confounding and dietary variables should be carefully considered. Nevertheless, these results are consistent with findings from Western countries [60,61] and warrant further investigation in Middle Eastern populations. For instance, a prospective study that examined the association between potato and French fry consumption and T2DM from the Nurses’ Health Study (NHSs) found a correlation between higher intake of potato products and the risk of T2DM [61]. Although the case-control study [35] suggested an association between fish consumption and the risk of diabetes, this may be attributed to the cooking style of fish in Saudi Arabia, as fish is usually deep fried and accompanied by either fried rice or bread and a fat dense sauce. Midhet *et al.* also highlighted the protective effect of increased vegetable consumption. A recent meta-analysis found that green leafy vegetables significantly decrease the risk of T2DM, while other vegetables have a modest protective effect [60].

Two recent cross-sectional studies [37,48] included in this review found that reduced intakes of animal protein and the use of NHVO are associated with a lower prevalence with T2DM in Middle Eastern adults. Despite the cross-sectional design, these findings are consistent with reports from other regions of the world. For example, the Adventist Health Study-2, that included 60,903 participants, found that semi-vegetarians had a lower risk of diabetes [62], and the Nurses’ Health Study reported an inverse association between vegetable fat and T2DM in women [63].

Observational evidence demonstrates mixed results on the association between DED and health outcomes [64]. However, nutritional studies suggest that DED is associated with a higher risk of diabetes [12] and diabetes markers [13,65]. Two cross-sectional studies in this review examined the association between DED and T2DM, however, the investigators used different methods to calculate DED. Although it has recently been suggested that calculating DED from food only is a better method to identify dietary risk factors for obesity [66], DED_Food_ failed to show an association, while DED_Food + Beverages_ seems to be associated with a higher risk of diabetes. The main limitation was the use of the FFQ as it largely underestimates caloric intake, and showed low correlations with energy biomarkers [67]. The results should be interpreted cautiously given the methodological limitations.

Although dietary patterns analysis lacks stability and includes some arbitrary decisions such as food grouping, this approach provides new insights of diet-disease relationship as it examines the diet as whole [14,68]. This method showed promising results in identifying dietary patterns associated with chronic conditions [69,70]. The lack of consistent associations between selected dietary patterns and T2DM in some of the included studies may be attributed to the lack of stability of dietary patterns, cross-sectional design and methodological limitations of the studies. Higher DDS [43], MD scores [42] and traditional Lebanese dietary patterns, which share similar aspects of the Mediterranean diet, seem to have a protective role against diabetes [44], which is in agreement with international evidence [71,72].

The intervention studies [50,52] included a limited number of clusters and data were reported at the individual level. The interventions were multi-factorial, so it is not possible to tease out the effects of dietary modifications alone. Nevertheless, the results show that at a population level such interventions are feasible, and whilst taking into account the methodological limitations, the results are promising, and in line with international evidence [22,73,74,75,76].

There are several limitations of the data included in this review that should be considered. The applicability of findings from the included studies, which originate from few countries in the Middle East, to other Middle Eastern populations is questionable, because dietary habits do vary across Middle Eastern countries [1,77]. Hence, there is a need for additional investigations, which might detect specific dietary factors associated with the risk of diabetes across different populations in the Middle East. The included studies were also of variable methodological quality. Data from cross-sectional analyses cannot infer causality, but can only describe associations between dietary variables and the prevalence of diabetes. Failing to control for confounding variables (*i.e.*, energy intake), and reverse-causation might undermine the validity of findings in some of the included studies. This could explain, for example, the paradoxical associations of green tea consumption with T2DM as reported in one of the included studies [39].

The FFQ is an inexpensive tool and easy to administer, it is widely used in nutritional epidemiological studies [78]. However, this nutritional tool is prone to the risk of recall bias [79], measurement error, and inaccuracy in assessing dietary intake. Nevertheless, in order to report accurate data, the validation of the FFQ is essential [4]. Only seven studies administered a validated FFQ, while two studies assessed diet using a 24-h dietary recall. The interpretation of results of the other seven studies is problematic, as some used non-validated tools and others administered questionnaires of poor structure and design.

T2DM is a growing public health problem in the Middle East [80]. Nutritional evidence recommends modifying diet composition to prevent T2DM [81,82]. Large-scale randomised trials have demonstrated that multifaceted lifestyle interventions, including dietary modification, represents an effective strategy in the prevention of T2DM especially in high-risk individuals [19,21,22,23,74,75,76,83,84], however this has not been confirmed in Middle Eastern populations. The evidence to date from the Middle East highlights the urgent need for well-designed dietary interventions, as very few studies have quantified the nutritional problem accurately [30,32,85]. Lack of validated nutritional tools across countries in the Middle East and the difficulty in assessing and reporting dietary intake accurately are possible factors for the scant body of nutritional evidence in the Middle East.

## 6. Conclusions

Despite extensive searching, relatively few studies met the inclusion criteria for this systematic review examining the association between dietary factors and risk of T2DM in Middle Eastern populations. Eating habits are obviously heterogeneous across different populations in the Middle East; nevertheless, they are likely to play an important role in the emerging epidemics of diabetes and obesity, or in other words, the “diabesity epidemic” [86] in the Middle East. Currently, the available data are not sufficient to identify specific dietary components associated with the risk of T2DM in these populations, and well-designed nutritional studies are needed.
